# National report on aggressions to physicians in Spain 2010–2015: violence in the workplace—ecological study

**DOI:** 10.1186/s13104-018-3393-7

**Published:** 2018-06-04

**Authors:** The National Observatory of Aggressions to Physicians (ONAM) Workgroup, J. M. Garrote-Díaz, A. Becerra-Becerra, J. Bendaña-Jácome, L. Casero-Cuevas, G. Garrote-Cuevas, M. Muñoz-García, R. Marín-Montero, L. Perez-Gallego, D. Gutierrez-Bejarano, J. R. Repullo, General Council of Official Medical Associations of Spain (CGCOM)

**Affiliations:** General Council of Official Medical Associations of Spain (CGCOM), Plaza de las Cortes, 11, 28014 Madrid, Spain

**Keywords:** Aggression, Physician–patient relations, Workplace violence

## Abstract

**Objective:**

Aggressions against health staff is a phenomenon that is not widely studied worldwide. To date, there is no national study analyzing this situation in Spain. Our objective is to describe and analyze aggressions to physicians of the whole Spanish territory in the period 2010–2015, through an observational analytical study by conglomerates (ecological) with all aggressions to physicians identified by the 52 official medical associations of Spain over 6 years of study.

**Results:**

There were 2419 aggressions on physicians, 51% on men. Primary care is the area that concentrates more incidents (54%) and the public sector is the most affected (89%). A third of the assaults were concentrated on professionals aged 46–55 years old. Cumulative incidence decreased from 20 aggressions × 10,000 physicians in 2010 to 15 × 10,000 physicians in 2015. The importance and seriousness of the problem of aggressions against physicians is verified through notifications to the registry. The collection method is different from others based on surveys, and therefore the figures are significantly lower than other studies. The scant denunciation by attacked physicians in Spain makes deceiving the real dimensions of the phenomenon.

## Introduction

Aggressions against physicians is a problem of growing interest in research, which has been poorly prioritized in healthcare systems. To date and knowledge of the authors, there is no regulated study in Spain that analyzes this situation and its trend at national level in recent years. The number of related international studies is limited, most of them are old, without evidence of a prospective analysis of the phenomenon and with disparate results, which may well be the indirect reflection of the characteristics of different health care systems and organizations, or of the particular situation of each country [1].

Our objective is to contribute to the current state of knowledge of this problem in Spain and serve as a reference in the European region.

Until 2015 there are very few published studies related to this phenomenon in our society [20]. In 2015 a survey of physicians in Barcelona [2] described how 44% of the professionals surveyed stated that they had been victims of some form of verbal aggression. Another regional survey, this time carried out in Aragon and Albacete to 1845 health professionals concluded that up to 64% of the sample had suffered aggressions of different magnitude, and 5% acknowledged having been attacked on multiple occasions [3].

## Main text

### Methodology

The study has an observational design, and analyzes both the set of cases accumulated in 6 years, and its longitudinal evolution. It collects all the aggressions on physicians registered by the National Observatory of Aggressions to Physicians (ONAM) of the CGCOM from 1st January 2010 to 31st December 2015. The population under study (N = 2419) is composed by of all physicians whose aggressions were registered by the 52 Official Medical Associations of Spain during this period. Each Medical Association reports its aggressions to the ONAM in a grouped manner, describing the scope of the professional practice in which they occur and the sociodemographic and professional characteristics of the physicians assaulted in each period. Considering that the general report of the ONAM is based on reports by clusters, the present study is ecological. The evaluation was anonymised.

#### Aggressions

We consider as an aggression each of the cases comprising physical or psychic attacks, insults or threats, which is communicated by the affected physicians to their respective Medical Association. We consider cluster, the report of aggressions of each Association to the ONAM. We have not excluded any of the detected aggressions.

#### Other variables

The information of the professionals assaulted on gender, age groups, type of professional exercise, presence of injuries, generation of (paid) sick leave (SL), reception of support/guidance by the company, presentation of a complaint, presence of previous aggressions and presence of material damages, and the information of the aggressor on personal background and profile, were obtained from the Annual Reports of the ONAM. Age variable was categorized into ranges (< 35, 36–45, 46–55, 56–65, ≥ 66 years).

The type of professional practice was categorized as public and private. The scope of the aggression was categorized as primary care (GP practices), hospital, primary care emergencies (out of hours and domiciliary care), hospital accident-emergencies services, and others. The aggressor’s personal history was categorized as drug addiction, psychiatric, organic disease and others. The profile of the aggressor was categorized as a scheduled patient, unscheduled patient, center user and family member. The other variables were dichotomized (yes/no) according to their presence or absence: personal injury, generation of temporary incapacity for work, reception of support/guidance by the company towards the physician attacked, presentation of a complaint, presence of previous aggressions and presence of material damages.

#### Statistical analysis

The descriptive analyses were based on frequency distribution and were performed on the total of aggressions observed in the study period. In the frequency analysis of each variable, only have been excluded the aggressions with lost data. For the accumulated incidence of aggressions, we used the number of assaults recorded each year, among the total number of collegiate doctors in the same period. P values were calculated using Pearson Square Chi or Fisher’s Exact Test. All statistical analyses were performed using Stata software (version 11.1) [4].

### Results

During the study period, there were 2419 aggressions on doctors in Spain, 50.8% on men and 49.2% on women. The great majority of the aggressions were detected in the public exercise (88.7%). 37.3% of incidents were concentrated in the age group 46–55 years. Table 1 shows the frequency distribution of the main characteristics of the population.

**Table 1:** Characteristics of the studied population 2010–2015

Characteristics	Distribution
Gender (n = 2401), %	
Men	1219 (50.8)
Women	1182 (49.2)
Type of practice (n = 2342), %	
Public	2078 (88.7)
Private	264 (11.3)
Age groups (n = 1809), years (%)	
≤ 35	254 (14.0)
36–45	494 (27.3)
46–55	675 (37.3)
56–65	353 (19.5)
≥ 66	33 (1.8)
Area (n = 1523), %	
Primary care	1267 (53.9)
Hospital	341 (14.5)
Out-of-hospital emergency rooms	225 (9.6)
Hospital emergency room	215 (9.1)
Other areas	303 (12.9)
Aggressor background (n = 1385), %	
Toxicomany	108 (7.8)
Psychiatric	184 (13.3)
Organic disease	169 (12.2)
Unknown	924 (66.7)
Aggressor profile (n = 1859), %	
Scheduled patient	604 (32.5)
Non scheduled patient	451 (24.3)
Center user	274 (14.7)
Relative or companion	530 (28.5)
Personal injuries (n = 2346), %	
Yes	465 (19.8)
No	1881 (80.2)
Sick leave (n = 2154), %	
Yes	263 (12.2)
No	1891 (87.8)
Company support (n = 1697), %	
Yes	544 (32.1)
No	1153 (67.9)
Formal complaint (n = 2283), %	
Yes	1655 (72.5)
No	628 (27.5)
Previous aggressions (n = 2179), %	
Yes	168 (7.7)
No	2011 (92.3)
Material damage (n = 2043), %	
Yes	213 (10.4)
No	1830 (89.6)

Values are expressed as absolute numbers and weighted percentages for categorical variables

#### Cumulative incidence

The cumulative incidence of aggressions against physicians in the period studied shows a significant tendency to decrease (P trend < 0.0001), observing a cumulative incidence of 20.2 assaults per 10,000 physicians in 2010 and 15 assaults per 10,000 physicians in 2015 (no significant differences between men and women).

#### Aggressions by area

Primary care concentrated more than half of the cases (53.9%), followed by the hospital and Accident and Emergency Services (23.6%).

#### Severity of injuries

Almost a fifth of the assaults on professionals during the study period were associated with personal injuries. Up to 12% of all attacks resulted in a SL. In 1 out of every 10 cases, material damages occurred and almost 8% of the physicians attacked had suffered prior attacks (Table 1). In a gender-stratified analysis of the aggressions recorded in 2015, no statistically significant differences were found in the proportion of physical and psychological injuries among men and women (Table 2).

**Table 2:** Comparison of the aggressions to physicians by area and gender 2015

Area	Men	Women	P value	Total
PC^a^ (%)	90 (50.3)	103 (56.6)	NS^d^	193 (58.3)
Hospital (%)	34 (19.0)	32 (17.0)	NS^d^	66 (18.3)
PUCC^b^ (%)	15 (8.4)	20 (11.0)	NS^d^	35 (9.7)
HER^c^ (%)	19 (10.6)	9 (4.9)	NS^d^	28 (7.8)
Other areas (%)	21 (11.7)	18 (9.9)	NS^d^	39 (10.8)

Values are expressed as absolute numbers and weighted percentages for categorical variables

^a^Primary Care

^b^Urgent Care Centers

^c^Hospital Emergency Rooms

^d^No significance

#### Aggressor profile

Only 7 out of 10 aggressions are done by the patient; the others are generated by a relative or companion. Regarding the aggressor’s antecedents, we found that 13% of aggressors had a history of psychiatric pathology, 12% of organic pathology and 8% of drug abuse; however, in the remaining 67% their medical history was unknown. It was significantly more frequent to find aggressions among the scheduled patients than among the non-programmed ones (32.5% vs 24.3%, P < 0.0001).

Causes related to aggressions: A cause could be identified in 1883 cases (78%) of the total number of assaults recorded in the period. The discrepancies of the aggressor with the medical care provided motivated one of every three attacks and became the most frequent cause. Other aspects related to the medical act itself and the decision making by the professional, such as the prescription of medicines, the issuance of temporary in capacity, or the generation of reports (11.9, 5.8 and 5.5% respectively) accounted for almost a quarter of the reported aggressions. Organizational aspects (waiting time, internal functioning, etc.) accounted for 15.4% of attacks (Table 3).

**Table 3:** Identified causes of aggressions to physicians in Spain 2010–1015

Cause related to the aggression	2010 N (%)	2011* N* (%)	2012 * N* (%)	2013 * N* (%)	2014 * N* (%)	2015 * N* (%)	Total * N* (%)
Discrepancies with healthcare	99 (31.3)	166 (39.3)	113 (35.5)	93 (32.1)	81 (30.8)	98 (35.8)	650 (34.5)
No prescription of desired medicine	38 (12.0)	56 (13.3)	44 (13.8)	33 (11.4)	25 (9.5)	29 (10.6)	225 (11.9)
Waiting time	36 (11.4)	37 (8.89	35 (11.0)	34 (11.7)	34 (12.9)	37 (13.5)	213 (11.3)
Personal discrepancies	31 (9.8)	25 (5.9)	34 (10.7)	25 (8.6)	30 (11.4)	42 (15.3)	187 (9.9)
Related to work incapacity	21 (6.6)	24 (5.7)	19 (6.0)	22 (7.6)	15 (5.7)	8 (2.9)	109 (5.8)
Medical reports that are not in accordance with the patient’s requests	23 (7.3)	17 (4.0)	11 (3.5)	25 (8.6)	13 (4.9)	15 (5.5)	104 (5.5)
Disagreement with the functioning of the centre	13 (4.1)	16 (3.8)	13 (4.1)	11 (3.8)	12 (4.6)	12 (4.4)	77 (4.1)
Other causes	55 (17.4)	81 (19.2)	49 (15.4)	47 (16.2)	53 (20.2)	33 (12.0)	318 (16.9)
Total	316	422	318	290	263	274	1883 (100)

We describe the sample size in each period. Values are expressed as absolute numbers and weighted percentages for categorical variables

#### Procedures after aggressions

In only one-third of the attacks, the doctor received some support or advice from his institution. However almost 73% filed a complaint with the competent authorities.

### Discussion

The phenomenon of aggressions against doctors during their professional practice is a latent phenomenon in many countries. However, in Spain, the number of published studies that analyze and quantify this situation is limited. A systematic review by Vidal-Marti et al. in 2014 [5] found only 16 articles on assaults in Spain published since 2000. And of these, only 6 analyze with quality the frequency of these events. At the international level, a systematic review by Nelsen et al. [6], finds only 12 studies published until 2013 that describe the prevalence of aggressions against physicians, but with results with a wide range (1.5–68.5%).

Our results allow us to see incidences of aggressions against physicians, ranging from 15 to 20 assaults per 10,000 doctors in the period studied. These numbers are significantly lower than those described by different authors.

The area in which we found more aggressions was primary care with 54% of incidents, a frequency very close to 58% described in other Spanish studies [7–9] and to 63% described in Anglo-Saxon studies [10]. Although we have not standardized the incidence rates of each of the areas studied, the greater proportion of professionals specialized in Family Medicine and the greater number of activities performed annually in primary care, we consider that explain the greater proportion of aggressions in this area.

In our data, gender was not a differentiating factor in the incidence of aggressions, as described by multiple studies [11–16]. However, in the analysis of differences by gender and type of aggression, there are studies such as that by Miedema et al. describe a higher frequency of sexual harassment-related aggressions among women than among men (60.7% vs 30.5%, P < 0.01) [17].

Although it has not been possible to calculate the rates of aggression by age group due to the absence of records, as it would have been necessary, it may be useful to make some comments on the distribution of age frequencies among the aggressed physicians. The age group with the highest proportion of recorded attacks was between 46 and 55 years old, a range higher than that described in other Spanish studies (30–43 years) [14–16, 18, 19]; but closer to the most affected ages described in international studies: Canada (44 years) [17], Australia (45–54 years old) [20] and Germany (55 years old) [17]. That is, our results support the hypothesis that the profile of the physician attacked is not necessarily a young physician with little experience as other authors have suggested [15, 21].

Low level aggressions (insults, threats) are not valued as serious by doctors in many cases, which is why they do not ask for help from the Medical Association and only communicate it to the contracting institution. Therefore the aggressions reported to the Medical Association may have a greater severity (Berkson selection bias): one out of every five aggressions had personal injuries and one out of every ten generated a SL and presented material damages. In fact, in other Spanish studies, the reported prevalence of physical damage is lower [3, 8, 9, 18, 19, 22].

Concerning the aggressor, it has been possible to identify his antecedents in a third of the cases, being the antecedents psychiatric and of drug addiction the most frequent, findings that coincide with those described by other authors [23–25].

### Conclusions

Aggressions against health professionals are a severe problem with important consequences for any health system. The real magnitude has been silenced by the low claims of assaulted physicians. It is a global phenomenon, which is present not only in other European countries (Germany, France, Great Britain, Norway), but also in other continents. Although it is a multicausal situation, the loss of respect for the doctor, especially in primary care and in the emergency rooms has favored an increase in violent attitudes. Although the studies show that a large proportion of physicians have suffered from some aggression in their professional practice, only a small proportion formalizes the complaint to the competent authorities.

It is decisive, within the clinical management, to take measures aimed at the prevention of aggressions and the attention to injured professionals, always starting from an individualized analysis of the work environment and the factors that can favor such situations. For this, it is important to maintain and develop research on this phenomenon.

Achieving the intervention of legislative and executive authorities, as it is already happening in Spain, for the recognition of the health professional as an authority [25, 26], can be the long way to go through other environments where the frequency of these events is increasing.

## Limitations

- Although the ONAM reports have a national character, as authors we are aware that the cases of aggressions reported to medical associations usually coincide with cases of greater severity, so that there may be an important under-registration of aggressions of less severity that never get to be reported by the professionals involved, being that the main limitation of our work. For our analysis, this can lead us to underestimate the real magnitude of the problem, justifying the low incidences found, lower than those described by other authors. That fact therefore limits the validity of the data provided and invites cautious interpretations of the results.
- As authors, we would like to point out that this is the first analytical study to explore ONAM consolidated information throughout Spain, and although its results are not directly comparable with other studies related to methodological differences, the analysis provides relevant data on the characteristics and typology of the aggressions to physicians, and constitutes a Spanish and European referent for future investigations.

## Abbreviations

ONAM: Observatorio Nacional de Agresiones a Médicos (National Observatory of Aggressions to Physicians)
SL: sick leave
